# Cerebellar anodal tDCS does not facilitate visuomotor adaptation or retention

**DOI:** 10.1016/j.brs.2022.10.006

**Published:** 2022-10-28

**Authors:** Caroline R. Nettekoven, Rebecca Jurdon, Tulika Nandi, Ned Jenkinson, Charlotte J. Stagg

**Affiliations:** Wellcome Centre for Integrative Neuroimaging, FMRIB, Nuffield Department of Clinical Neurosciences, University of Oxford, OX3 9DU, UK; Oxford Centre for Human Brain Activity, Wellcome Centre for Integrative Neuroimaging, Department of Psychiatry, University of Oxford, OX3 7JX, UK; Medical Research Council Brain Network Dynamics Unit, Nuffield Department of Clinical Neurosciences, University of Oxford, OX1 3TH, UK; Department of Psychiatry, University of Oxford, OX3 7JX, UK; Wellcome Centre for Integrative Neuroimaging, FMRIB, Nuffield Department of Clinical Neurosciences, University of Oxford, OX3 9DU, UK; Wellcome Centre for Integrative Neuroimaging, FMRIB, Nuffield Department of Clinical Neurosciences, University of Oxford, OX3 9DU, UK; Oxford Centre for Human Brain Activity, Wellcome Centre for Integrative Neuroimaging, Department of Psychiatry, University of Oxford, OX3 7JX, UK; Medical Research Council Brain Network Dynamics Unit, Nuffield Department of Clinical Neurosciences, University of Oxford, OX1 3TH, UK; Johannes Gutenberg University Medical Centre, Germany; Johannes Gutenberg University Medical Centre, Germany School of Sport, Exercise and Rehabilitation Sciences, University of Birmingham, B15 2TT, UK; Wellcome Centre for Integrative Neuroimaging, FMRIB, Nuffield Department of Clinical Neurosciences, University of Oxford, OX3 9DU, UK; Oxford Centre for Human Brain Activity, Wellcome Centre for Integrative Neuroimaging, Department of Psychiatry, University of Oxford, OX3 7JX, UK Medical Research Council Brain Network Dynamics Unit, Nuffield Department of Clinical Neurosciences, University of Oxford, OX1 3TH, UK

**Keywords:** Cerebellum, tDCS, Neuromodulation, Adaptation, Retention

## 1 Introduction

Adaptation of our movements is crucial to retain motor accuracy in a changing environment. Appropriate motor adaptation relies on cerebellar activity [1–4], and the application of cerebellar anodal transcranial direct-current stimulation (atDCS) during lab-based visuomotor adaptation task has been reported to facilitate error reduction in young healthy subjects [5–7] and in older adults [8,9]. This beneficial behavioural effect appears to extend to other adaptation paradigms including force-field adaptation [10], locomotor adaptation [11] and saccadic adaptation [12]. However, recent studies have reported no effect of cerebellar tDCS on joystick visuomotor adaptation [13,14](but see Ref. [15]) and the original study [5] has been difficult to replicate and revealed only small-to-medium effect sizes [13].

Here, we wished to replicate the facilitatory effect of cerebellar atDCS on visuomotor adaptation. To increase the likelihood of observing an effect of cerebellar stimulation, we used a within-subject design and a novel adaptation task paradigm designed to bias learning towards primarily cerebellum-dependent processes [16,17]. In this task, a stepwise increasing rotation of 10° every 40 trials required participants to adapt movements to reduce errors.

## 2 Methods

Twenty-seven healthy, right-handed [18] participants (17 female, 18–32 years) gave informed consent (Oxford University R53867/RE001) to participate in a within-subject, double-blind study. Participants received cerebellar anodal and sham tDCS in two sessions (order counterbalanced across the group) while performing a visuomotor adaptation task on separate days. Participants had no contraindications to tDCS, no neurological or psychiatric history, and no neuroactive medications.

**Fig. 1:**
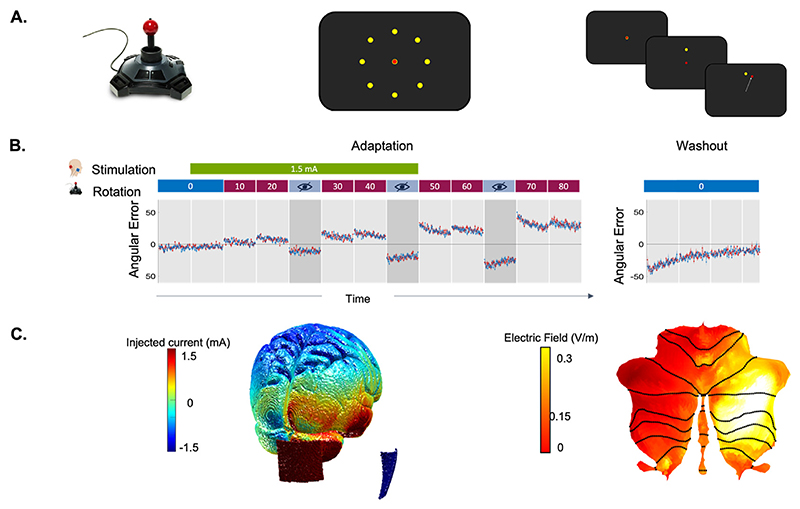
Results. A. Visuomotor Adaptation Task. Participants used a joystick to shoot targets on a screen. Targets appeared in one of eight possible locations radially aligned around the centre starting position, separated by 45° (middle panel). After 80 baseline trials, the visual feedback of the cursor was rotated by 10°. This resulted in a 10° offset between joystick movement and cursor movement (right panel). Over time, participants learned to adapt to this offset. B. No effect of cerebellar tDCS on visuomotor adaptation or retention. Participants adapted to a visuomotor rotation while receiving sham or real anodal tDCS to the cerebellum. Participants shot targets on a screen. They began by performing 80 trials with no rotation imposed serving as the baseline in both conditions. After the baseline, stepwise increasing rotated visual feedback (red blocks), required participants to adapt movements to reduce errors. One block at each angle and each block consisted of 40 trials of 4 seconds duration each. The numbers in the red and blue boxes indicate the degree to which the visual feedback was rotated, with 0° indicating no rotation. The imposed rotation reached a maximum of 80°. Interspersed blocks in which visual feedback was removed (crossed out eye) served to probe retention of the adapted movement. The rotation was washed out after task (144 trials, no rotation). Behavioural data is shown as angular error at each trial averaged across participants. Shaded area represents standard error of the mean. There was no difference in angular error between the real condition (red) and sham condition (blue). C. Electrical Field Magnitude in cerebellum. The injected current (left) and mean electrical field magnitude (right) induced by cerebellar anodal tDCS in a representative participant. The distribution of the electrical field magnitude in the cerebellum is shown on the SUIT flatmap [20]. The electric field induced in the brain was estimated using SimNIBS 3.2.3. The head model was built from the representative subject’s T1-weighted structural image using the standard headreco pipeline. The cerebellum grey and white matter segmentation estimated using headreco was replaced by a more refined segmentation obtained using CERES, after visual inspection revealed superior segmentation results from CERES [21]. The anode was centered on the right cerebellar cortex, 3 cm right of the inion, in line with the pre-auricular point. The cathode was positioned over the right buccinator muscle. (For interpretation of the references to colour in this figure legend, the reader is referred to the Web version of this article.)

Participants controlled a cursor on a screen using a joystick and made fast, accurate and ballistic movements to ‘shoot’ through a target at one of eight possible locations aligned around the central starting position (Fig. 1A). The cursor remained visible and no specific endpoint feedback was provided. Each movement had to be completed within 750 ms, after which the target disappeared. Participants performed 80 baseline trials (no rotation imposed) followed by 8 blocks of 40 trials each with an initial offset between joystick and cursor movement of 10°, increasing a further 10° every 40 trials to a maximum of 80° (Fig. 1B). To probe retention, three blocks with no visual feedback were interspersed, after each of which participants had a 20 second break. On completing the main task participants performed 144 baseline trials.

A DC-Stimulator (Neuroconn GmbH, Ilmenau, Germany) delivered a 1.5 mA (0.06 mA/cm2) current via two 5 × 5 cm electrodes (Easycap, Germany) using Ten20® conductive paste. The anode was centered on the right cerebellar cortex, 3 cm right of the inion, in line with the pre-auricular point, and the cathode was centered over the right buccinator muscle [5] (Fig. 1C). Real stimulation delivered a 1.5 mA current for 20 minutes (green block in Fig. 1B). Sham stimulation delivered 1.5mA for 30 seconds (10 second ramp up/down) at the beginning and end of the 20 minute stimulation period[52]. At the end of each session, participants reported any adverse sensations. Both participant and experimenter independently recorded the perceived stimulation condition.

Cursor movements were analyzed on a trial-by-trial basis using in-house software written in Matlab (Mathworks Inc, Natick, USA). Joystick position was sampled at 60Hz, filtered with a zero-phase filter (25Hz cut-off), and numerically differentiated to determine velocity. For each trial, the angular error (°) was defined as the angle between a line connecting the starting position with the position of peak velocity of the cursor and the line connecting the starting position with the target. Positive values indicate a clockwise error (‘overshooting’). Trials with premeditated or otherwise poorly-defined movements were excluded from further analysis (12.3 ± 12.2[mean ± SD] trials (0.02 ± 0.02%) excluded per subject).

Adaptation error was calculated as the mean error across all trials excepting the first 8 of each rotation block, to capture the rapid error reduction of adaptation performance excluding the initial exposure to the rotation [5,19]. Adaptation error was calculated for the stimulation period alone (Fig. 1B: green block) and for the full task period (Fig. 1B: red blocks). Retention was calculated as the mean error in all blocks with no visual feedback [5] (Fig. 1B: pale blue blocks). Linear mixed models were constructed with stimulation and session as fixed effects and a varying intercept for subject. P-values were obtained by likelihood ratio tests of the full model with the effect in question against the model without the effect.

## 3 Results

Both participant and experimenter blinding was successful (Correctly identified current condition: Participant 37%, Experimenter 48%). Baseline performance did not differ between sessions (1 v 2: χ^2^(1) = 0.58, p = 0.45), nor between real and sham sessions (χ^2^(1) = 2.15, p = 0.14). Baseline performance did not differ during real and sham stimulation (χ^2^(1) = 1.36, p = 0.24).

As expected, participants learned to compensate for the imposed rotation across the experiment (Initial eight v last eight trials t(53) = 5.5, p < 0.001). However, participants performed better in Session 2 than Session 1 across the task (χ^2^(1) = 4.54, p = 0.03), though this improvement was smaller in the stimulation period alone (χ^2^(1) = 2.85, p = 0.09). We therefore included session as a covariate of no interest into our model in all further analyses. Adaptation performance did not differ significantly between real and sham stimulation either during stimulation (χ^2^(1) = 1.05, p = 0.3) or across the whole task (χ^2^(1) = 0.15, p = 0.7). Finally, to avoid any session effects, we tested for an effect of stimulation in the first session only. Adaptation performance did not differ significantly between subjects receiving real and sham stimulation in the first session (F(1,25) = 0.23, p = 0.64).

Here, we wanted to probe implicit mechanisms of adaptation. Awareness of any offset between the joystick and the cursor might increase the use of explicit strategies, potentially engaging different neural substrates [17]. When asked, 13 participants remained unaware of any perturbation. Analysing this small subset of participants, a trend towards real stimulation improving adaptation was seen (χ^2^(1) = 3.22,p = 0.07) but this could be explained by baseline differences (χ^2^(1) = 12.3, p > 0.1).

We saw no effect of stimulation on retention, either across the whole experiment (χ^2^(1) = 1.62, p = 0.2) or during the stimulation phase (χ^2^(1) = 0.9, p = 0.34).

## 4 Discussion

Potential explanations for our failure to replicate a significant behavioural effect of cerebellar tDCS, include significant between- session differences. However, given our sample size, and the null finding when only considering the first session, at the most the behavioural effect of cerebellar tDCS appears much smaller than previously reported [5].

While session order effects pose a difficulty for motor adaptation experiments, within-subject studies are crucial in controlling for the physiological variability in tDCS studies. Our results highlight the critical need to investigate the physiological, brainstate and task-dependent factors influencing tDCS to reconcile conflicting evidence on behavioural effects of cerebellar tDCS. An investigation into the precise physiological changes in the cerebellum in response to tDCS could help navigate the vast parameter space and further the development of cerebellar tDCS for clinical applications.

## Data Availability

Behavioural data is available at: https://github.com/carobellum/cerebellar_tdcs_adaptation. MRI data will be shared on the data sharing platform of the Wellcome Centre for Integrative Neuroimaging which is currently under development. In the interim, MRI data is available upon request.
